# Diversity of functional edaphic macrofauna in *Musa acuminata x Musa balbisiana* (AAB) agroecosystems

**DOI:** 10.12688/f1000research.127300.1

**Published:** 2022-11-14

**Authors:** C. A. Zúniga-Gonzalez, A. J. Caballero-Hernández

**Affiliations:** 1 Department of Agroecology, Faculty of Agrarian and Veterinary Sciences, Bioeconomy and Climate Change Research Center, National Autonomous University of Nicaragua, Leon, 21000, Nicaragua; 2 Research Department, Quality Assurance and Post-harvest Technology Section, Del Monte Agricultural Development Corporation, S. A. PINDECO, Provincia Limón, Cantón Pococí Distrito Guápiles, 70101, Costa Rica

**Keywords:** Eco-Intensification, Eco-system, Plantain, Diversity, Microfauna

## Abstract

**Background:** This study focused on evaluating the diversity and richness of the edaphic macrofauna in eight banana farms in the western zone of Nicaragua.

**Methods:** The sampling design was random and descriptive, it was divided into two phases, the first was the collection of the sample, and the second was the classification, coding, and storage of the extracted macrofauna populations. Subsequently, the indices of diversity and species richness, relative abundance, by functional groups were estimated.

**Results:** The results showed that the relative abundance of biodiversity was higher in the 0–20 cm soil depth stratum than in the branch and leaf biomass strata. The values of the diversity indices of Dominion, Simpson, Shanon, Margalef, and Equity were in the normal range, with a tendency towards low diversity. Likewise, in the richness of species, the Dominant or most abundant genus were earthworms (Oligochaeta) and Hymenoptera ( *Solenopsis*, *Leptothorax*, *Camponotus*, *Pheidole*), indicating the directly proportional relationship, that is to say, that the greater the number of earthworms the production increases and the greater the number of Hymenoptera it decreases, confirmed with the Pearson correlation coefficient with a reliability of 95%.

**Conclusions:** It was concluded that based on the estimates of the diversity indicators, two detritivore genus (earthworms and Hymenoptera) were the ones with the greatest presence, being important in the production of the banana agrosystem due to the decomposition of organic matter and its nutritional contribution to the plant. We observed a direct correlation with earthworms and an indirect relationship with Hymenoptera.

## Introduction

Soil management in banana agroecosystems is essentially successful for resilient agro-ecological production, adapted to climate change, and biodiversity, fundamentally in Latin America. Berning *et al.* (2022), Gliessman (2013), Rousseau *et al.* (2012), McLaughlin *et al.* (1995), Challinor *et al.* (2009), Rousseau *et al.* (2013) and Delgado *et al.* (2010b) indicate that agro-ecological conditions of the soil represent one of the ways to adapt precisely to the development of production in the banana sector. The combination of *Musa acuminata × Musa balbisiana* (AAB) constitutes a food source in the Latin American diet ( Belalcázar, 2003). For this reason, it is important to develop agricultural practices linked to the benefit of edaphic biodiversity and thus have productive and intensive agriculture characterized by various degrees of intensification of traditional, customary, transitional and organic agriculture ( Delgado *et al.*, 2010a). Improving ecosystems and taking advantage of the usefulness of biodiversity in this process requires understanding the structure and function of biological and physical-chemical combinations. They include stability of the edaphic structure, reuse, storage, and supply of organic matter (OM) and nutrients, available soil moisture, and management of damage to micro, and macrofauna.

Similarly, authors such as McKelvie-Sebileau *et al.* (2022), Samudio (2010), Shiyam *et al.* (2010), López (1995), MIFIC (2007) and Gizzi *et al.* (2009) suggest that in nutritional irrigation systems where crops such as corn, beans, pumpkin, papaya, pineapple, coffee, cocoa are included, they demand the conservation of fungi, bacteria, viruses, harmful insects, nematodes and weed organisms, to do more effective environmental perceptions and producer profitability.

Likewise, Gutiérrez-Luna *et al.* (2022), Rodríguez *et al.* (2013) and Brown *et al.* (2001) investigated that the impact of cultural practices or soil health should be evaluated through chains that maintain soil fertility. From this perspective, soil fertility studies of plantain should include previously unexplained constituents of the macrofauna as indicators of the richness in biodiversity, abundance, and degree of alteration of the ecological functions of the population.

On the other hand, invertebrate pests attract a lot of attention, and they cost farmers and producers millions of dollars ( Tresson *et al.*, 2022; Velásquez *et al.*, 2012; Alcaraván, 2003; Decaëns *et al.*, 2004; Cardona *et al.*, 1998).

Brown *et al.* (2001) and Pocasangre, Brown and Quesada (2009) show that limited physical-chemical elements and fertility can lead to population decline. In the same way, they added that beneficial invertebrates for their basic and fundamental functions have received little attention. In general, Velasquez *et al.* (2007) shows that their behavior is taken for granted and the management of agroecosystems is rarely altered for their benefit. The importance of the invertebrate edaphic macrofauna is closely related to the quantitative and qualitative analysis of biomass period (MO) and generation of genetic biology components.

Medina *et al.* (2021), Djigal *et al.* (2012), Laossia *et al.* (2008) and Ruiz (2008) indicate that these organisms can experience a shortage of oxygen and light, fewer open spaces, poor availability and quality of food and a very strong variability of microclimates to be able to live in the soil, examples of these microorganisms are the centipedes, termites, earthworms, insects, mites, flying worms and butterflies ( Medina *et al.*, 2021). Populations of all megafaunas reach millions per hectare and their biomass varies in tons per hectare. Their diversity can exceed 1,000 species in complex ecosystems (such as tropical forests), but precise data on the specific diversity of tropical edaphic megafauna in specific ecosystems is still lacking ( Anderson, 1993; Zerbino, 2010; Zerbino *et al.*, 2008; Priego-Castillo *et al.*, 2009; Wardle *et al*., 1995).

Finally, the work focused on evaluating the diversity and abundance of macrofauna in eight banana-producing farms in the North Pacific area of Nicaragua. The work was organized in an introductory section where the problem and the importance of this study are explained. In the Methods section, the procedure for the collection, classification, coding and storage of the species is presented. The Results and Discussion section presents the species found and their richness, abundance and diversity. Finally, the main conclusions based on the objective of the research are presented.

## Methods

Table 1 shows the statistical description of the data used in this study. The full protocol can be found on protocols.io.

**Table 1.** Descriptive statistics.

Farm	N	Minimum	Maximum	Mean	Std. Deviation	Variance
Farm1	30	0.00	150.00	17.3333	35.30003	1246.092
Farm2	30	0.00	325.00	18.6667	61.99184	3842.989
Farm3	30	0.00	50.00	4.8333	12.14022	147.385
Farm4	30	0.00	65.00	7.5000	17.75067	315.086
Farm5	30	0.00	150.00	12.6667	33.18530	1101.264
Farm6	30	0.00	225.00	17.1667	52.50096	2756.351
Farm7	30	0.00	155.00	11.8333	33.20478	1102.557
Farm8	30	0.00	125.00	15.0000	34.56628	1194.828

Source: Self calculation' authors.

### Geographic location of the study

**Figure 1.**
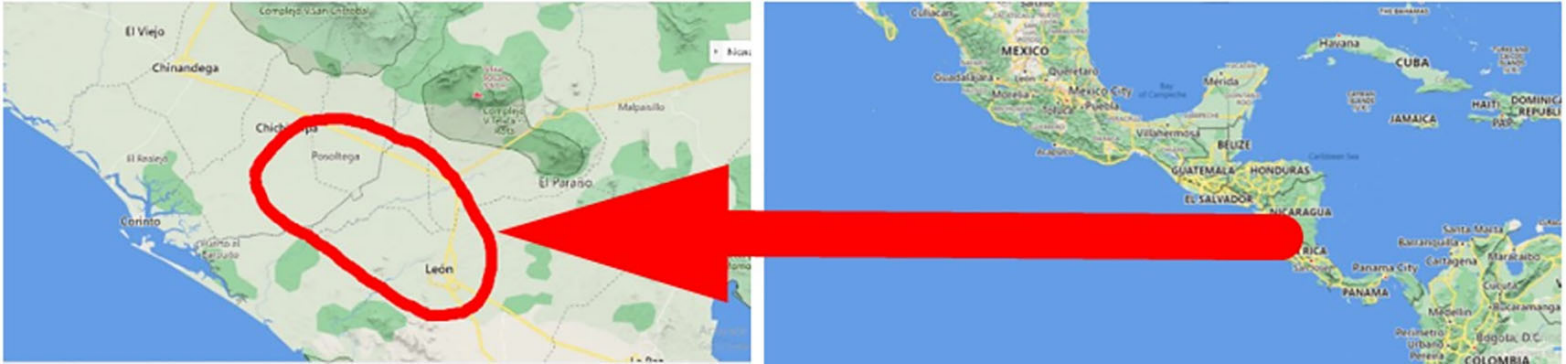
Study area. Map data ©2022 Google.

The study was carried out in eight banana farms in the city of León and Posoltega ( Table 2). The climatic conditions in the León area are characterized by having a rainfall of 1,529.7 mm, an average temperature per year of 38°C, and an altitude of 60 meters. The Posoltega area is characterized by an average annual temperature of 39°C, 2,000 mm of rain and an altitude of 70.42 meters above sea level, both areas are located in the western region of Nicaragua, see Figure 1 ( MIFIC, 2007).

**Table 2.** Location of banana farms in the study area.

ID farmer	Farm name	Area	Community	Municipality	Latitude	Longitude
1	Santa Isabel	266	Rio Grande N° 3	León	12,664,471	-86,848,814
2	Quinta Cony	15.2	San Pedro	León	12,664,471	-86,848,814
3	San Martin	72.2	San Pedro	León	12,664,471	-86,848,814
4	El Verdón	7.6	San Pedro	León	12,664,471	-86,848,814
5	San Joaquín	4.6	El Trianon	Posoltega	12521742	-86,994,189
6	Montes Verdes	2.3	Chiquimulapa	Posoltega	12,535,643	-86,981,141
7	María de los Ángeles	3.8	El Trianon	Posoltega	12521742	-86,994,189
8	Los Ángeles	5.3	El Trianon	Posoltega	12,521,742	-86,994,189

Source: Self calculation' authors.

### Investigative process phase

Table 2 shows eight farms that describe the name, area, community and municipality. In these farms, the investigation begins with the field phase where 40 edaphic samples of 0–20 cm depth and 40 biomass samples (leaf litter) were collected on the surface. A total of 80 samples of macrofauna populations were identified, coded, stored in the second phase. The study area in each farm was 0.7 ha delimited 1 in 1,000 m ^2^ (50 cm long × 20 cm wide), as described in Rousseau *et al.* (2012), Rousseau *et al.* (2013), Medina *et al.* (2021) *.*

After selecting the sampling area, sampling points are placed to collect soil samples. A wooden box 20 cm wide by 20 cm long was used to mark the sampling points, and to remove approximately 1 kg of soil. Divided into two consecutive layers (fallen leaves, 0–20 cm), each of them is surrounded to prevent microorganisms from escaping from the bottom, after which the material is sieved and separated manually and the insects found are placed in an airtight plastic bottle. After measuring 500 cubic centimeters in volume, they are labeled and preserved in 70% alcohol.

### Identification of macrofauna species

The collected individuals were analyzed by order, family and quantified and identified by sex, the microorganisms were placed in Petri dishes and then observed under a 4–400× stereo microscope, to detail the specific structures of each of the species. Large animals include all organisms greater than 4 mm in length.

### Measured variables

The diversity and richness of species present in this study were analyzed using the indicators reported by the authors Zerbino (2010) and Rousseau *et al.* (2012, 2013). Richness (number of species): the number is the number of species for each farm, which was determined and totaled for each sampled system. The total number of individuals per species was counted and estimated.

### Functional groups (various population densities 1 m ^2^)

Population abundance and species richness were determined by three main functional groups: herbivores, detritivores, and predators. To estimate the density, PAST (RRID:SCR_019129) 4.03 software was used, the indices selected for the study were: Domain (D), Shannon-Wiener (H′), Margalef (Mg), Simpson (1-D), and Pielou (J′).

Domain Index (D): It is the relative importance of a species related to the degree of influence it has on the individuals of the plantain agrosystem. It is based on competition for resources, which is why the characterization of the collected sample is used and then organized by functional group. Its inverse is the Simpson index.

Shannon-Wiener Index (H′) ( Equation 1): Considers the number of species found in the study area (species richness) and the relative frequency (abundance) of each of these species. It is used to determine the number of species and how those species are distributed. It is usually expressed as H′, expressed as a positive number that varies between 0.5 and 5. Values between 0.5–2 indicate a situation with low diversity, 2–3 is a normal situation, and 3–5 or more indicates a situation of high diversity.

$$H^{\prime }=-\sum p_{i}\mathrm{Ln}p_{i}$$ (1)

*S*: Richness or number of species; *pi*: ratio of individuals of the species ( *i*) with respect to the total number of individuals (that is, the relative abundance of the species *i*).

Margalef Diversity Index (DMg) ( Equation 2): measures the specific richness of an area and the relationship between individuals and the total sample. The value 0 is when there is only one species in the sample ( *s*=1, therefore *s*-1=0), values less than 2 are considered areas of low biodiversity and values greater than 5 are indicative of high biodiversity. Where:

Dmg=S−1LnN (2)

*S* = number of species; *N* = total number of individuals.

Simpson's Index (1-D) ( Equation 3): this index is based on dominance. This is the inverse parameter of the concept of community unity or equality. Consider the representativeness of the most important species without evaluating the contribution of the remaining species. Values from 0 to 0.5 bring the value closer to a situation of high diversity, and values from 0.5 to 1 bring the value closer to low biodiversity.

Where:

$$\lambda =\sum p_{i}^{2}$$ (3)

*P*_*i*_ = the number of individuals among the total species ( *i*). Strongly influenced by the importance of the dominant species. Its value is the reciprocal of fairness, so the diversity is known as 1 – λ.

Pielou Index ( *J*) ( Equation 4): stock market index. It measures the relationship between the observed diversity and the maximum expected diversity. Its value is between 0 and 0.1, so 0.1 corresponds to situations in which all species occur equally.

Where:

J′=H′Hmax′ (4)

### Data analysis

Using IBM SPSS Statistics (RRID:SCR_016479) v.22 program, the data of individuals collected and ordered by categories were processed. Tables were created showing the groups of species present in each farm studied. For the analysis of the diversity indices, the PAST (RRID:SCR_019129) 4.03 software was used. Finally, Pearson's correlation was applied to identify the most dominant and most common group useful to understand the dominant interrelationship and its productivity within the plantain agrosystem.

## Results and Discussion

### Relative and dominant abundance

**Figure 2.**
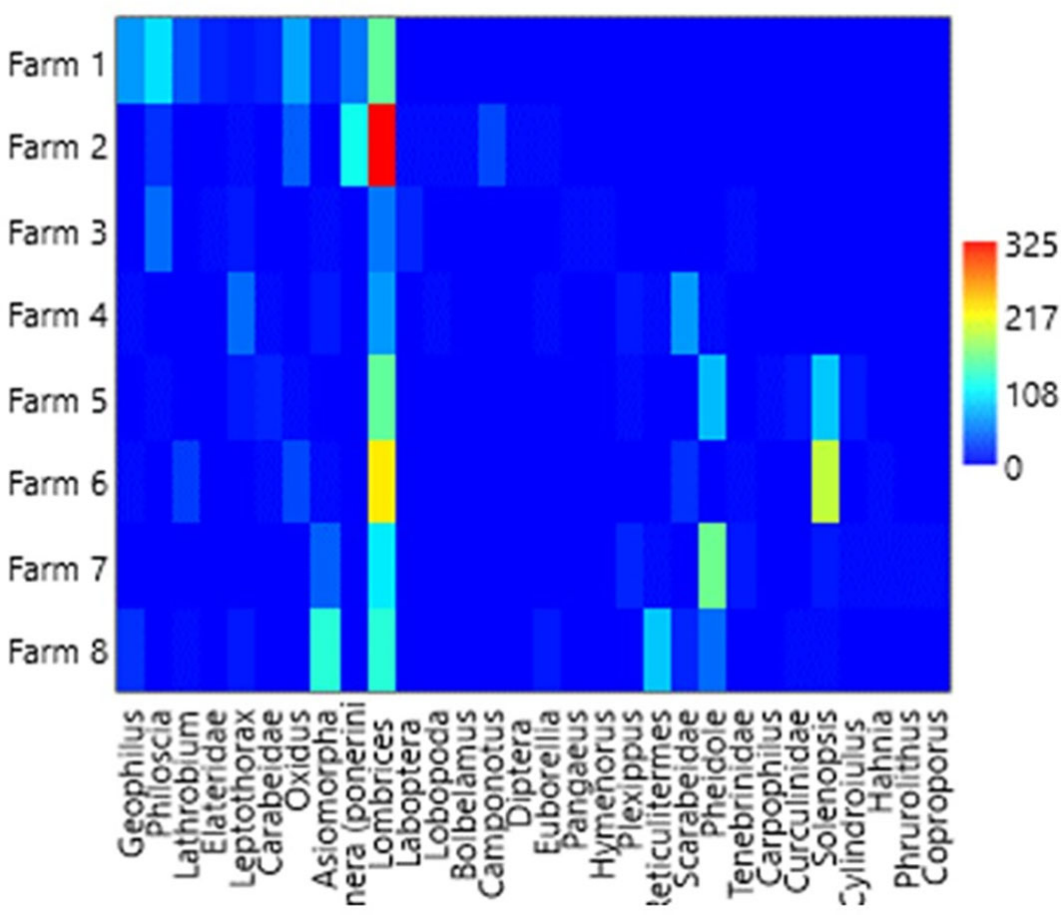
Overall relative abundance of macrofauna found. This heatmap was constructed using PAST software.

Tables 1, 3 and 4, and Figure 2 ( Zuniga-Gonzalez *et al.*, 2022) show the general relative abundance of macrofauna found in the four banana plantations in the city of León between the litter layer and soil depths of 0–20 cm. The genera *Geophilus* and *Leptothorax* dominate with 70 individuals per square meter (ind × m ^2^), *Philoscia* with 160 ind × m ^2^ and *Oxidus* with 110 ind. m ^2^ and *Hypoponera* are 165 ind × m ^2^, and earthworms are 590 ind × m ^2^. In the municipality of Posoltega, *Pheidole* sp. 280 ind × m ^2^, *Solenopsis* sp. 290 ind × m ^2^, *Asiomorpha* 170 ind × m ^2^, earthworm 600 ind × m ^2^ ( Tables 5 and 6). The genus *Pheidole* sp., was found in the soil of a banana plantation in the city of León, and the genus *Solenopsis.* However, the frequencies of earthworm individuals are similar in both communities. Zerbino *et al.* (2008) in their study presented the two most abundant groups of the subclass Oligochaeta of the order Opisthopora and insects of the order Hymenoptera, representing 46 and 20% of the total number of individuals collected, respectively. This confirms our findings with earthworms ( *Opisthopora*) and Hymenoptera ( *Pheidole* and *Solenopsis*) accounting for 37.77 and 18.25%, respectively, of all individuals collected.

**Table 3.** Total relative abundance ( *p*_*i*_) according to functional group by farms.

Species number	Genus	Leon municipality								Posoltega municipality								Total %	
		Farm 1	*p*_*i*_	Farm 2	*p*_*i*_	Farm 3	*p*_*i*_	Farm 4	*p*_*i*_	Farm 5	*p*_*i*_	Farm 6	*p*_*i*_	Farm 7	*p*_*i*_	Farm 8	*p*_*i*_	Biomass	*p*_*i*_
HERBIVORES																			
1	*Diptera*	0.00	0.00	5.00	0.50	0.00	0.00	0.00	0.00	0.00	0.00	0.00	0.00	0.00	0.00	0.00	0.00		
2	*Elateridae*	15.00	0.60	0.00	0.00	5.00	0.33	0.00	0.00	0.00	0.00	0.00	0.00	0.00	0.00	0.00	0.00		
3	*Leptothorax*	10.00	0.40	5.00	0.50	10.00	0.67	45.00	1.00	10.00	1.00	0.00	0.00	0.00	0.00	10.00	1.00		
	*Total*	25.00	1.00	10.00	1.00	15.00	1.00	45.00	1.00	10.00	1.00	0.00	0.00	0.00	0.00	10.00	1.00	115.00	0.04
	*p*_*i*_		0.05		0.02		0.10		0.20		0.03		0.00		0.00		0.02		
DETRITIVORES																			
4	*Philoscia*	95.00	0.26	20.00	0.05	45.00	0.38	0.00	0.00	5.00	0.03	0.00	0.00	0.00	0.00	0.00	0.00		
5	*Lathrobium*	35.00	0.10	0.00	0.00	0.00	0.00	0.00	0.00	0.00	0.00	25.00	0.08	0.00	0.00	5.00	0.01		
6	*Oxidus*	70.00	0.19	40.00	0.10	0.00	0.00	0.00	0.00	5.00	0.03	30.00	0.10	0.00	0.00	0.00	0.00		
7	*Asiomorpha*	15.00	0.04	0.00	0.00	5.00	0.04	10.00	0.07	0.00	0.00	5.00	0.02	40.00	0.24	125.00	0.35		
8	*Lombrices*	150.00	0.41	325.00	0.81	50.00	0.42	65.00	0.43	150.00	0.88	225.00	0.73	100.00	0.61	125.00	0.35		
9	*Laboptera*	0.00	0.00	5.00	0.01	15.00	0.13	0.00	0.00	0.00	0.00	0.00	0.00	0.00	0.00	0.00	0.00		
10	*Lobopoda*	0.00	0.00	5.00	0.01	0.00	0.00	5.00	0.03	0.00	0.00	0.00	0.00	0.00	0.00	0.00	0.00		
11	*Bolbelamus*	0.00	0.00	5.00	0.01	0.00	0.00	0.00	0.00	0.00	0.00	0.00	0.00	0.00	0.00	0.00	0.00		
12	*Reticulitermes*	0.00	0.00	0.00	0.00	0.00	0.00	5.00	0.03	0.00	0.00	0.00	0.00	5.00	0.03	85.00	0.24		
13	*Scarabeidae*	0.00	0.00	0.00	0.00	0.00	0.00	65.00	0.43	0.00	0.00	20.00	0.06	0.00	0.00	15.00	0.04		
14	*Darkling beetle*	0.00	0.00	0.00	0.00	5.00	0.04	0.00	0.00	0.00	0.00	5.00	0.02	10.00	0.06	0.00	0.00		
15	*Cylindroiulus*	0.00	0.00	0.00	0.00	0.00	0.00	0.00	0.00	10.00	0.06	0.00	0.00	5.00	0.03	0.00	0.00		
16	*Coproporus*	0.00	0.00	0.00	0.00	0.00	0.00	0.00	0.00	0.00	0.00	0.00	0.00	5.00	0.03	0.00	0.00		
	*Total*	365.00	1.00	400.00	1.00	120.00	1.00	150.00	1.00	170.00	1.00	310.00	1.00	165.00	1.00	355.00	1.00	2035.00	0.65
	*p*_*i*_		0.70		0.71		0.83		0.67		0.45		0.60		0.46		0.79		
PREDATORS																			
17	*Geophilus*	65.00	0.50	0.00	0.00	0.00	0.00	5.00	0.17	0.00	0.00	5.00	0.02	0.00	0.00	20.00	0.24		
18	*Pheidole*	0.00	0.00	0.00	0.00	0.00	0.00	5.00	0.17	80.00	0.40	0.00	0.00	155.00	0.82	45.00	0.53		
19	*Carabeidae*	15.00	0.12	0.00	0.00	0.00	0.00	5.00	0.17	15.00	0.08	5.00	0.02	0.00	0.00	0.00	0.00		
29	*Hypoponera (ponerini)*	50.00	0.38	115.00	0.77	0.00	0.00	0.00	0.00	0.00	0.00	0.00	0.00	0.00	0.00	0.00	0.00		
21	*Camponotus*	0.00	0.00	30.00	0.20	0.00	0.00	0.00	0.00	0.00	0.00	0.00	0.00	0.00	0.00	0.00	0.00		
22	*Euborellia*	0.00	0.00	5.00	0.03	0.00	0.00	5.00	0.17	0.00	0.00	0.00	0.00	0.00	0.00	10.00	0.12		
23	*Pangaeus*	0.00	0.00	0.00	0.00	5.00	0.50	0.00	0.00	0.00	0.00	0.00	0.00	0.00	0.00	0.00	0.00		
24	*Hymenorus*	0.00	0.00	0.00	0.00	5.00	0.50	0.00	0.00	0.00	0.00	0.00	0.00	0.00	0.00	0.00	0.00		
25	*Plexippus*	0.00	0.00	0.00	0.00	0.00	0.00	10.00	0.33	5.00	0.03	0.00	0.00	15.00	0.08	0.00	0.00		
26	*Carpophilus*	0.00	0.00	0.00	0.00	0.00	0.00	0.00	0.00	5.00	0.03	0.00	0.00	0.00	0.00	0.00	0.00		
27	*Curculinidae*	0.00	0.00	0.00	0.00	0.00	0.00	0.00	0.00	10.00	0.05	0.00	0.00	0.00	0.00	5.00	0.06		
28	*Solenopsis*	0.00	0.00	0.00	0.00	0.00	0.00	0.00	0.00	85.00	0.43	190.00	0.93	10.00	0.05	5.00	0.06		
29	*Hahnia*	0.00	0.00	0.00	0.00	0.00	0.00	0.00	0.00	0.00	0.00	5.00	0.02	5.00	0.03	0.00	0.00		
30	*Phrurolithus*	0.00	0.00	0.00	0.00	0.00	0.00	0.00	0.00	0.00	0.00	0.00	0.00	5.00	0.03	0.00	0.00		
	Total	130.00	1.00	150.00	1.00	10.00	1.00	30.00	1.00	200.00	1.00	205.00	1.00	190.00	1.00	85.00	1.00	1000.00	0.32
	*p*_*i*_		0.25		0.27		0.07		0.13		0.53		0.40		0.54		0.19		
Total *S* and *p*_*i*_		520.00	3.00	560.00	3.00	145.00	3.00	225.00	3.00	380.00	3.00	515.00	2.00	355.00	2.00	450.00	3.00		
															Grand total			3150.	1.00

Source: Self calculation' authors.

**Table 4.** Total relative abundance according to functional group in the farms of the municipality of León.

Functional group	Farm 1	Farm 2	Farm 3	Farm 4	Farm 5	Farm 6	Farm 7	Farm 8	Total
Herbivores	5	2	10	20	3	0	0	2	4
Detritivores	70	71	83	67	44	60	46	79	64
Predators	25	27	7	13	53	40	54	19	32

Source: Self calculation' authors.

**Table 5.** Total population of species collected in litter strata and 0-20 cm ^2^ soil depth.

Species number	Species	Leon municipality					Posoltega municipality				
		Farm 1	Farm 2	Farm 3	Farm 4	Total, Leon	Farm 5	Farm 6	Farm 7	Farm 8	Total, Posoltega
1	*Geophilus*	65	0	0	5	70	0	5	0	20	25
2	*Philoscia*	95	20	45	0	160	5	0	0	0	5
3	*Lathrobium*	35	0	0	0	35	0	25	0	5	30
4	*Elateridae*	15	0	5	0	20					0
5	*Leptothorax*	10	5	10	45	70	10	0	0	10	20
6	*Carabeidae*	15	0	0	5	20	15	5	0	0	20
7	*Oxidus*	70	40	0	0	110	5	30	0	0	35
8	*Asiomorpha*	15	0	5	10	30	0	5	40	125	170
9	*Hypoponera (ponerini)*	50	115	0	0	165	0	0	0	0	0
10	*Lombrices*	150	325	50	65	590	150	225	100	125	600
11	*Laboptera*	0	5	15	0	20	0	0	0	0	0
12	*Lobopoda*	0	5	0	5	10	0	0	0	0	0
13	*Bolbelamus*	0	5	0	0	5	0	0	0	0	0
14	*Camponotus*	0	30	0	0	30	0	0	0	0	0
15	*Diptera*	0	5	0	0	5	0	0	0	0	0
16	*Euborellia*	0	5	0	5	10	0	0	0	10	10
17	*Pangaeus*	0	0	5	0	5	0	0	0	0	0
18	*Hymenorus*	0	0	5	0	5	0	0	0	0	0
19	*Plexippus*	0	0	0	10	10	5	0	15	0	20
20	*Reticulitermes*	0	0	0	5	5	0	0	5	85	90
21	*Scarabeidae*	0	0	0	65	65	0	20	0	15	35
22	*Pheidole*	0	0	0	5	5	80	0	155	45	280
23	*Tenebrinidae*	0	0	5	0	5	0	5	10	0	15
24	*Carpophilus*	0	0	0	0	0	5	0	0	0	5
25	*Curculinidae*	0	0	0	0	0	10	0	0	5	15
26	*Solenopsis*	0	0	0	0	0	85	190	10	5	290
27	*Cylindroiulus*	0	0	0	0	0	10	0	5	0	15
28	*Hahnia*	0	0	0	0	0	0	5	5	0	10
29	*Phrurolithus*	0	0	0	0	0	0	0	5	0	5
30	*Coproporus*	0	0	0	0	0	0	0	5	0	5

Source: Self calculation' authors.

**Table 6.** Taxonomic classification of the edaphic macrofauna in kingdom, phylum, subphylum, class, order, family and genus.

Species number	Kingdom	Phylum	Class	Order	Family	*Genus*	Functional group
1	Animalia	Arthopoda	Insecta	Diptera		*Diptera Linneaeus, 1758*	Herbivores
2	Animalia	Arthopoda	Insecta	Coloptera	Elateridae	*Elateridae*	Herbivores
3	Animalia	Arthopoda	Insecta	Hemenoptera	Formiciae	*Leptothorax Mayr, 1855*	Herbivores
4	Animalia	Arthopoda	Malacostraca	Isopoda	Philosciidae	*Philoscia Latreille, 1804*	Detritivores
5	Animalia	Arthopoda	Insecta	Coloptera	Staphylinidae	*Lathrobium Gravenhorst, 1802*	Detritivores
6	Animalia	Arthopoda	Diplopoda	Polydesmida	Paraxosomatidae	*Oxidus Cook, 1911*	Detritivores
7	Animalia	Arthopoda	Diplopoda	Polydesmida	Paraxosomatidae	*Asiomorpha coarctata Verhoeff, 1939*	Detritivores
8	Animalia	Annelida	Clitellata	Opisthopora	Lumbricidae	*Lombrices*	Detritivores
9	Animalia	Annelida	Insecta	Blattoidea	Blattidae	*Laboptera sp*	Detritivores
10	Animalia	Annelida	Insecta	Coleoptera	Tenebrionidae	*Lobopoda Soller, 1835*	Detritivores
11	Animalia	Arthopoda	Insecta	Coleoptera	Bolboceratidae	*Bolbelamus*	Detritivores
12	Animalia	Arthopoda	Insecta	Isotera	Rhinotermitidae	*Reticulitermes Holmgren, 1913*	Detritivores
13	Animalia	Arthopoda	Insecta	Coleoptera	Scarabaeidae	*Scarabaeidae Latreille, 1802*	Detritivores
14	Animalia	Annelida	Insecta	Coleoptera	Tenebrionidae	*Darkling beetle*	Detritivores
15	Animalia	Arthopoda	Diplopoda	Julida		*Cylindriuslus terutonicus Pocock, 1900*	Detritivores
16	Animalia	Arthopoda	Insecta	Coleoptera	Staphylinidae	*Coproporus*	Detritivores
17	Animalia	Arthropoda	Chilopoda	Geophilomorpha	Geophillidae	*Geophilus Leach, 1814*	Predators
18	Animalia	Arthopoda	Insecta	Hymenoptera	Formocidae	*Pheidole Westwood, 1839*	Predators
19	Animalia	Arthopoda	Insecta	Coloptera	Carabidae	*Carabeidae*	Predators
20	Animalia	Arthopoda	Insecta	Hymenoptera	Formicidae	*Hypoponera Santschi, 1938*	Predators
21	Animalia	Arthopoda	Insecta	Hymenoptera	Formicidae	*Camponotus Mayr, 1861*	Predators
22	Animalia	Arthopoda	Insecta	Dermaptera	Anisolabididae	*Euborellia Burr, 1910*	Predators
23	Animalia	Arthopoda	Insecta	Hemiptera	Cydnidae	*Pangaeus*	Predators
24	Animalia	Arthopoda	Insecta	Coleoptera	Alleulidae	*Hymenorus*	Predators
25	Animalia	Arthopoda	Arachnida	Araneae	Salticidae	*Plexippus*	Predators
26	Animalia	Arthopoda	Insecta	Coleoptera	Nitidulidae	*Carpophilus Stephens,1830*	Predators
27	Animalia	Arthopoda	Insecta	Coleoptera	Curculionoidea	*Curculinidae*	Predators
28	Animalia	Arthopoda	Insecta	Hymenoptera	Formicidae	*Solenopsis Westwood, 1840*	Predators
29	Animalia	Arthopoda	Arachnida	Araneae	Hahnidae	*Hahnia C. L. Koch, 1841*	Predators
30	Animalia	Arthopoda	Arachnida	Araneae	Phrurolithidae	*Phrourolithus C.l Koch, 1839*	Predators

Source: Self calculation' authors.

In a study by Priego-Castillo *et al.* (2009) and Castillo and Vera (2000), it was reported that the Hymenoptera group was the most abundant, with 62.49% of the total individuals collected during the 2,000 agricultural cycle in organic and conventional banana plantations in Guacimo, Costa Rica. This means that the soil is moist throughout the year. However, the presence of earthworms ( *Opisthopora*) was 11.92% and abundant in all stages.

In the municipality of Leon, Farm 1 shows that earthworms dominate at 150 ind × m ^2^ and *Oxidus* dominate with 70 ind × m ^2^. In farm 2, earthworms dominate at 325 ind × m ^2^, and *Hypoponera* spp. with 115 ind × m ^2^, likewise in farm 4 El Verdón, stand out, genus *Leptoxthorax* (Opisthopora) of 45 ind × m ^2^, 65 ind × m ^2^ of Scarabaeidae and 65 ind × m ^2^ of earthworms, however, in farm 3, only the genus, *Philoscia*, in Farm 3, measures 45 ind × m ^2^ and earthworms dominate at 50 ind × m ^2^ ( Table 5 and 6). The municipal of León has a greater wealth of sexes with 23 representative genus, but a lower overall dominance (ind × m ^2^). In four farms (San Martín, Santa Isabel and El Verdon) earthworm frequencies were found to be similar, but less common genera such as *Leptothorax* and *Asiomorpha* were found.

These data on earthworm populations in litter and layers from 0 to 20 cm ^2^ have been confirmed by Castillo and Vera (2000), Pashanasi (2001), Zerbino (2010), and these populations have a beneficial role in the soil. Training is very important and sensitive to management practices in the banana farms of León and Posoltega.

Table 4 shows the relative abundance by farm and by functional group, noting that the detritivores group is more abundant, followed by predators. These dominant groups exerted a beneficial function on the soil, allowing increased production yields because they are the responsible for breaking down OM and providing nutrition for plants.

For the municipality Posoltega, in farm 6, earthworms dominate with 225 ind × m ^2^ and *Solenopsis* spp. with 190 ind × m ^2^, farm 8 had *Asiomorpha* spp. with 125 ind × m ^2^ ( Table 5 and 6). Plantain plantations in Posoltega are repopulated with *Solenopsis* worms and ants. This is likely because they occupy the same ecological niches in which they coexist, interact with similar food sources, land, husbandry and management skills, and benefit from abundant and prosperous communities. These data are supported by Zayas *et al.* (2022), Quiroz-Medina *et al.* (2021), Castillo y Vera (2000), Pashanasi (2001), Zerbino (2010), Rousseau *et al.* (2012) and Rousseau *et al.* (2013).

### Diversity indices

Table 7 shows the diversity indices per farm. In general, the farms studied present a taxonomic variety of biomass (3,150 individuals). In general, a low domain (less than 40%) is observed in each of the farms. The Shannon-Wiener index shows low diversity with values less than 2, except for farms 1, 4 and 8 with values close to 2, meaning normal diversity. The Shannon indexes are not superior to those of Melo (2010), who had a high diversity value of the Shannon index of H′ = 2.61, which indicates that the Kikuyu prairie has the highest richness in both families and organisms. Rousseau *et al.* (2012, 2013), present low-quality data on diversity, dominance, wealth, and stock market indices.

**Table 7.** Diversity index by farm.

Index	Farm 1	Farm 2	Farm 3	Farm 4	Farm 5	Farm 6	Farm 7	Farm 8
Taxa_S	10	11	9	11	11	10	11	11
Individuals	520	560	145	225	380	515	355	450
Dominance (D)	0.167	0.3887	0.2366	0.2138	0.2545	0.3347	0.287	0.2044
Simpson (1-D)	0.833	0.6113	0.7634	0.7862	0.7455	0.6653	0.713	0.7956
Shannon (H)	1.989	1.358	1.73	1.824	1.673	1.393	1.6	1.828
Margalef (Mg)	1.439	1.58	1.607	1.846	1.683	1.441	1.703	1.637
Equitability (J)	0.8637	0.5663	0.7873	0.7605	0.6975	0.6051	0.6671	0.7622

Source: Self calculation' authors.

The Simpson index confirms this low diversity with values close to 1. The specific richness of the area and the relationship between individuals and the total sample reflected by the Margalet index (DMg) with values less than 2. This is considered as areas of low biodiversity. The Pielou evenness index indicates that not all farms presented situations where all species were equally abundant ( Krebs, 1999).

### Functional groups

Table 3 shows a summary of the relative abundance of biodiversity in the study area by functional group ( Castañeda *et al.*, 2022; Quiroz-Medina, 2021). As mentioned above, the species equity indices are not equal. The lowest percentage of taxonomic presence is in the functional group of herbivores. The biomass has a greater presence (above 50% in the group by function of detritivores and in the group of predators below 50%).

In studies reported by Zerbino *et al.* (2008) and Zerbino (2010), it is shown that the discrepancies in the constitution of the megafauna’s community and proportions of functional groups are aspects influenced by the species, the richness of plant species and management, and states that it affects living organisms. This is because they determine the available resources and influence the interactions between herbivores, their controllers, and the destroyers identified by Moore *et al.* (2004).

This supports the findings regarding the fact that monoculture influences the available resources and, therefore, is capable of affecting the interactions between functional groups × m ^2^ in the city of Posoltega. The results indicated that the texture of the biocenosis is consistent with the edaphic properties and the quantity and quality of the residues ( Almonte, 2022; Leyva, 2012; Curry, 1992). In zero tillage, management practices that promote the presence of residues with spatial and temporal diversification of plant species have richer, more diverse and equitable communities, with a predominance of deterioration functional groups ( Priego *et al.*, 2009; Zerbino, 2010; Zerbino *et al.*, 2008). This is consistent with the findings in the present study.

The analysis confirmed that Posoltega had lower populations of herbivores per m ^2^ and 460 carnivores per m ^2^ ( Table 3).

Table 8 shows the diversity indicators by functional groups. These are predominantly detritivores and predators of 400 × m ^2^, in contrast to El Verdón on farm 4, which has a low population of herbivores of 400 × m ^2^, detritivores 85 ind. × m ^2^ due to the large population of predators found. Similarly, at Farm 8 Los Angeles there were no herbivores. This is due to the large population of 250 ind. × m ^2^ predators ( Tables 3- 5 and 6).

**Table 8.** Diversity Indexes by functional group.

Indexes	Farm 1	Farm 2	Farm 3	Farm 4	Farm 5	Farm 6	Farm 7	Farm 8
**Herbivores**								
Taxa_S	2	2	2	1	1	0	0	1
Individuals	25	10	15	45	10	0	0	10
Dominance (D)	0.52	0.5	0.5556	1	1	0	0	1
Index (1-D)	0.48	0.5	0.4444	0	0	0	0	0
Index (H)	0.673	0.6931	0.6365	0	0	0	0	0
Index (Mg)	0.3107	0.4343	0.3693	0	0	0	0	0
Index (J)	0.971	1	0.9183	0	0	0	0	0
**Detritivores**								
Taxa_S	5	6	5	5	4	6	6	5
Individuals	365	400	120	150	170	310	165	355
Dominance (D)	0.2843	0.6731	0.3333	0.3822	0.7837	0.5473	0.4325	0.3073
Index (1-D)	0.7157	0.3269	0.6667	0.6178	0.2163	0.4527	0.5675	0.6927
Index (H)	1.388	0.7131	1.257	1.132	0.4845	0.9716	1.135	1.271
Index (Mg)	0.678	0.8345	0.8355	0.7983	0.5841	0.8716	0.9793	0.6812
Index (J)	0.8627	0.398	0.7812	0.7034	0.3495	0.5423	0.6333	0.7898
**Predators**								
Taxa_S	3	3	2	5	6	4	5	5
Individuals	130	150	10	30	200	205	190	85
Dominance (D)	0.4112	0.6289	0.5	0.2222	0.35	0.8608	0.6759	0.3564
Index (1-D)	0.5888	0.3711	0.5	0.7778	0.65	0.1392	0.3241	0.6436
Index (H)	0.9632	0.639	0.6931	1.561	1.259	0.3422	0.713	1.262
Index (Mg)	0.4109	0.3992	0.4343	1.176	0.9437	0.5636	0.7623	0.9004
Index (J)	0.8768	0.5816	1	0.9697	0.7025	0.2468	0.443	0.7843

Source: Self calculation' authors.

The data collected in the León area show that the herbivorous functional group has a low proportion of Diversity by the values of the Shannon-Wiener index (H) with a value close to 1 denoting low diversity, this is considered within normality, Dominance Simpson (1-D) with a value close to 1 and Pielou Equity (J′) very close to 1 as in the case of farm 2, meaning equally abundant species. However, the Margalef Diversity Index (DMg) showed a value below 2 considering for areas of low diversity.

Three indicators dominate in the functional group of detritivorous organisms in the city of León. Margalef Diversity (DMg) with a value below 2 means that no farm with this group can be considered as having high diversity. For Simpson Dominance (1-D) it can be said that they are at a midpoint between 1 and 0 with a normal diversity and a Pielou Equity Index (J′) with a value close to 1 situation where all species are equally abundant, of the indices for the Predators group were dominated by three indices: DMg in farm 4, which almost reaches value 2, it can be said that they are within the normal range, and Shannon-Wiener Diversity (H) with values lower than 1 indicating low diversity. This contrasts with the Posoltega area, where Simpson (1-D) dominates with a value close to 0, which implies a high diversity.

Table 8 shows the estimates of the indices of population abundance and species richness, determined by functional groups. According to the DMg, the value found was less than 2 considered as a zone of low biodiversity, which assumes that the number of individuals is equal to the number of species. In the Simpson index, the probability that eight individuals taken at random are of the same species is 0.2 in the case of León and 0.19, this constitutes a low probability, since most of the farms are made up of the genus *Lombrices*, and that had a lot of abundance of the same species. The Pielou Equity Index (J) confirms these with values close to 1 where all species are equally abundant.

### Diversity and productivity

**Figure 3.**
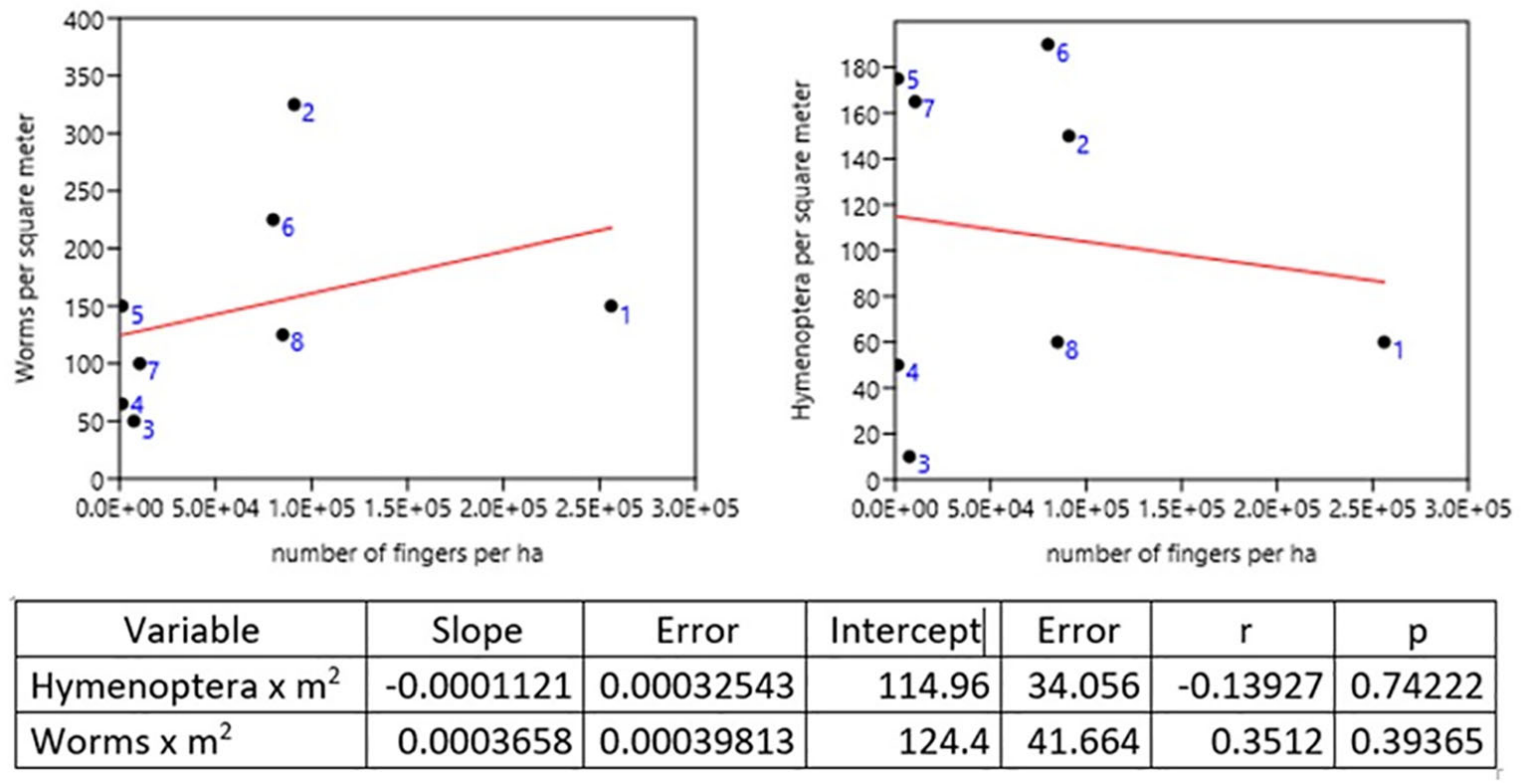
Real production *vs.* Hymenoptera and worms.

Samples were taken for the comparative study between plantain production and the two most abundant genera, earthworms and Hymenoptera, both of which are beneficial in the decomposition of OM and the supply of nutrients to the plant. In the dominance of worms, the Quinta Cony (Farm 2) was found where 325 ind × m ^2^ were collected, followed by the Montes Verde farm (Farm 6) with 225 ind × m ^2^. Hymenoptera were dominant on the San Joaquín farm (Farm 5) with 175 ind × m ^2^ and on farm 7 with 165 ind × m ^2^ ( Table 9 and Figure 3).

**Table 9.** Agricultural production (banana units per ha) and the abundance of earthworms and hymenopteran insects (ind × m ^2^).

Farm ID	Community	Real production of number of fingers per ha	Hymenoptera per square meter	Worms per square meter	Estimated overall earthworm production	Estimated production by zone of worms or Hymenoptera
1	Santa Isabel	256,000	60	150	38183.9	33585.36
2	Quinta Cony	91,000	150	325	96299.82	88084.08
3	San Martin	7,500	10	50	4974.8	2206.36
4	El Verdón	1,300	50	65	9956.16	1524.32
5	San Joaquín	1,250	175	150	38183.9	25825
6	Montes Verdes	80,000	190	225	63090.72	70250
7	María de los Ángeles	10,500	165	100	21579.35	4125
8	Los Ángeles	85,000	60	125	29881.62	85000

Source: Self calculation' authors.

**Figure 4.**
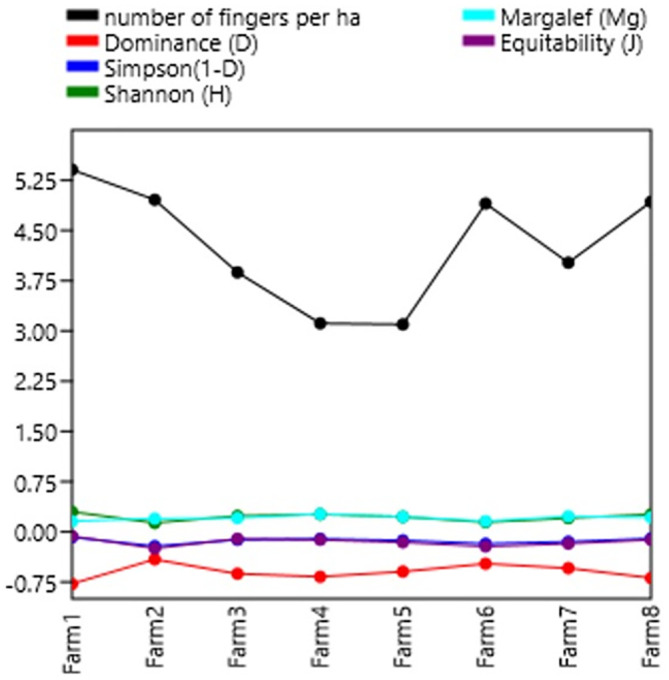
Relationship between diversity indices and productivity in plantain agroecosystems, Nicaragua.

To compare the psyllium production of each farm, the proportion of worms and insects of the two most abundant genera, the order Hymenoptera, was taken, indicating that a greater number of worms indicates a greater production. It was observed that for Hymenoptera, the production is lower. The farms with the highest production in the León area were Quinta Cony with 91,000 × ha and Santa Isabel with 25,600 × ha. The farm of 80,000 y × ha in farm 8 and farm 6 with 85,000 × ha in Montes Verdes for the Posoltega area ( Figures 3 and 4).

Pearson's correlation analysis commonly correlates agricultural production (plantain units per ha) and the abundance of earthworms and hymenopteran insects, projecting a correlation close to 1 with 95% confidence. The relationship between plantain production per ha and the abundance of earthworms of ind. × m ^2^ establishes a direct relationship, with a Pearson correlation coefficient of 0.743, close to 1, and a perfect or strong relationship in the Pearson correlation analysis ( Table 10). That is, by increasing the number of earthworms in the soil, the production of plantain per ha increases in the farms studied in both regions (León and Posoltega). On the other hand, a Pearson correlation coefficient of 0.261 ( Table 9) was obtained, which indicated that the relationship between production and abundance of Hymenoptera is very close to 0, thus showing a weak correlation between production and abundance of Hymenoptera insects.

**Table 10.** Pearson correlation coefficients of the production of Cuerno Dwarf plantain units (AAB) with the density per square meter of earthworms and Hymenoptera insects.

Study zone	Production	Lombrices	Hymenopteras
**Both areas**			
**Production**		***0.743*** *	0.261 *
**Lombrices**	***0.743*** *		0.608 *
**Hymenopteras**	0.261 *	0.608 *	
**León**			
**Production**		***0.988*** *	***0.942*** *
**Lombrices**	***0.988*** *		***0.968*** *
**Hymenopteras**	**0.942** *	**0.968** *	
**Posoltega**			
**Production**		0.465 *	-0.484 *
**Lombrices**	0.465 *		0.469 *
**Hymenopteras**	-0.484 *	0.469 *	

Source: Self calculation' authors.

* Significance p < 0.05.

The city of León has three strong ties. The first relationship (Pearson correlation coefficient 0.988) between productivity (psyllium units per ha on the farm) and earthworm abundance ( Table 10) shows a strong correlation. The second relationship between productivity and abundance of Hymenoptera insects has a Pearson correlation coefficient of 0.942 ( Table 10), indicating a strong correlation. A third relationship between the abundances of earthworms and hymenopteran insects is a Pearson correlation coefficient of 0.968 ( Table 10), which is very strong because the coefficient is close to 1, indicating that the number of earthworms becomes a direct relationship as it increases. In the municipality of Posoltega, the relationship between earthworm production and abundance is weak, with a Pearson correlation coefficient of 0.465. A Pearson correlation coefficient of -0.484 ( Table 10) indicates a negative relationship, with a decrease in the second abundance ratio of earthworms and hymenopterans. However, the abundance of hymenopteran insects is preserved. The increase in the number of worms increased the production and abundance of Hymenoptera, with a Pearson correlation coefficient of 0.469, favoring a direct relationship. On the other hand, the relationship between the number of hymenopteran insects and production is inversely correlated, with a Pearson correlation coefficient of -0.484 ( Table 10). That is, as the number of Hymenoptera decreases, production decreases ( Table 10). Table 11 shows the Pearson correlation coefficients by farm.

**Table 11.** Pearson correlation by farm.

Farm1	Farm2	Farm3	Farm4	Farm5	Farm6	Farm7	Farm8
1	.781 ^**^	.765 ^**^	.377 ^*^	.502 ^**^	.526 ^**^	.278	.407 ^*^
.781 ^**^	1	.667 ^**^	.539 ^**^	.704 ^**^	.686 ^**^	.433 ^*^	.520 ^**^
.765 ^**^	.667 ^**^	1	.426 ^*^	.513 ^**^	.477 ^**^	.302	.394 ^*^
.377 ^*^	.539 ^**^	.426 ^*^	1	.461 ^*^	.450 ^*^	.303	.462 ^*^
.502 ^**^	.704 ^**^	.513 ^**^	.461 ^*^	1	.863 ^**^	.747 ^**^	.518 ^**^
.526 ^**^	.686 ^**^	.477 ^**^	.450 ^*^	.863 ^**^	1	.368 ^*^	.428 ^*^
.278	.433 ^*^	.302	.303	.747 ^**^	.368 ^*^	1	.590 ^**^
.407 ^*^	.520 ^**^	.394 ^*^	.462 ^*^	.518 ^**^	.428 ^*^	.590 ^**^	1

Source: Self calculation' authors.

Correlation is significant at the *0.05 and **0.01 levels (2-tailed).

## Conclusions

The diversity and richness of the edaphic macrofauna was evaluated in eight farms in the western area. A total of 78.72% of the individuals were identified in the soil from 0–20 cm ^2^, while the remaining 21.26% in the foliage. In the first four farms in the León area, 23 genera were found, and in the Posoltega farms, 21 genera. The relative abundance of León was 1,450 individuals per m ^2^, while in the Posoltega area it was 1,700 individuals per m ^2^.

The Shannon-Wiener (H′) Diversity values were in the range of 1.6–1.9, which indicates a situation of low diversity, Margalef Diversity (D) obtained values between 1.4–1.8, lower than 2, considered as areas of low biodiversity, the Simpson's dominance (1-D) presented values between 1.4–1.8, considering that the closer it is to 1, a situation of low diversity is considered, and that of equality of Pielou (J′) presents 5-.8, so that the values close to 1 correspond to situations where all species are equally abundant. These results were due to the relative abundance of two detritivore genera (earthworms and Hymenoptera), which are organisms that decompose OM and provide nutrients to the plant.

Finally, it is concluded that these two genera are important in the production of the plantain agrosystem due to the decomposition of OM and its nutritional contribution to the plant, observing a direct correlation with earthworms and an indirect one with Hymenoptera.

## Data Availability

Zenodo: Data for: Diversity of Functional Edaphic Macrofauna in Musa acuminata × Musa balbisiana (AAB) Agroecosystems. https://doi.org/10.5281/zenodo.7242968 ( Zuniga-Gonzalez *et al.*, 2022). This project contains the following underlying data:
- DataLeon.csv (data for farms in Leon) - DataPosoltega.csv (data for farms in Posoltega) - Figue_2.jpg - Figure_1.jpg - Figure_3.jpg - Figure_4.jpg - NIH.csv (data on genera) - Tabla 3.png -
Table 4_.jpg -
Table 5.png -
Table 6.png - Table_1.jpg - Table_10.jpg - Table_11.jpg - Table_2.jpg - Table_7.jpg - Table_8.jpg - Table_9.jpg Data are available under the terms of the Creative Commons Attribution 4.0 International license (CC-BY 4.0).
