# Supplementary material for: Diversity of functional edaphic macrofauna in Musa acuminata x Musa balbisiana (AAB) agroecosystems
Source: F1000Res. 2024 Jun 18;11:1300. Originally published 2022 Nov 14. [Version 4] doi: 10.12688/f1000research.127300.4 (PMC11325132; doi:10.12688/f1000research.127300.4)
Supplement: Peer review history

# Reviewer report: Reviewer response for version 4

**DOI:** 10.5256/f1000research.164823.r309724

**Published:** 2024-08-14

**Authors:** Micaela Carvajal Alcaraz

**Affiliations:** 1 CEBAS-CSIC. Campus Universitario de Espinardo, Murcia, Spain

The study focuses on evaluating the diversity and richness of edaphic macrofauna in banana farms in Nicaragua. This is a relevant area of research as soil biodiversity plays a crucial role in agroecosystem productivity and sustainability. The specific focus on banana farms adds practical significance, considering the economic importance of bananas in tropical agriculture. This research presents a solid foundation and offers meaningful insights into soil biodiversity in banana farms. With a short additional detail and expanded discussion, it could serve as an important reference for both scientists and practitioners in the field of agroecology

 SMALL POINTS

- In one of the headings of the material and methods it is say ‘Identification of macrofauna species’, but the description includes “…the microorganisms were placed in Petri dishes and then observed under a 4–400× stereo microscope”.  Are macroorganism of microorganism? Please clarify.
- A deeper discussion on the implications of the findings for banana farm management and broader ecological impacts would add depth to the study. Just adding some sentences in the discussion and conclusion would be enough strength the manuscript.

Is the work clearly and accurately presented and does it cite the current literature?

Yes

If applicable, is the statistical analysis and its interpretation appropriate?

Yes

Are all the source data underlying the results available to ensure full reproducibility?

Yes

Is the study design appropriate and is the work technically sound?

Yes

Are the conclusions drawn adequately supported by the results?

Yes

Are sufficient details of methods and analysis provided to allow replication by others?

Yes

Reviewer Expertise:

Plant Sciences

I confirm that I have read this submission and believe that I have an appropriate level of expertise to confirm that it is of an acceptable scientific standard.

# Reviewer report: Reviewer response for version 3

**DOI:** 10.5256/f1000research.161242.r238299

**Published:** 2024-03-21

**Authors:** Jérôme Mathieu

**Affiliations:** 1 Sorbonne Universite, Paris, Île-de-France, France

My general comment is that a good effort has been done to improve the text and data availability, but there are still a few issues in the text, and most importantly, the claim that there is a strong relationship between taxa abundance and crop productivity is not supported by the data that are presented. This section needs to be changed. Please find detailed comments in the PDF linked here.

Is the work clearly and accurately presented and does it cite the current literature?

Yes

If applicable, is the statistical analysis and its interpretation appropriate?

Partly

Are all the source data underlying the results available to ensure full reproducibility?

Partly

Is the study design appropriate and is the work technically sound?

Yes

Are the conclusions drawn adequately supported by the results?

Partly

Are sufficient details of methods and analysis provided to allow replication by others?

Partly

Reviewer Expertise:

NA

I confirm that I have read this submission and believe that I have an appropriate level of expertise to confirm that it is of an acceptable scientific standard, however I have significant reservations, as outlined above.

# Response

**Published:** 2024-04-08

**Authors:** C. A. Zuniga-Gonzalez

**Affiliations:** Agroecology. ECAV. CIByCC., National Autonomous University of Nicaragua, Leon. Bioeconomy and Climate Change Research Center., Leon, Leon, Nicaragua

Thank you for your valuable feedback. We have thoroughly addressed all your comments and suggestions. Specifically, we have ensured the robustness of our data by applying bootstrap analysis to the correlation between production and the abundance of Hymenoptera and worms. This has strengthened the reliability of our findings and provided more insight into the relationship between agricultural productivity and soil fauna abundance. We appreciate your attention to detail and are confident that these enhancements significantly improve the quality of our study.

# Reviewer report: Reviewer response for version 1

**DOI:** 10.5256/f1000research.139795.r177964

**Published:** 2023-07-19

**Authors:** Jérôme Mathieu

**Affiliations:** 1 Sorbonne Universite, Paris, Île-de-France, France

**Main comments**

- Please improve the English language and grammar.
- Please give more information about the differences between the plots and use them to explain the differences in soil macrofauna communities.
- Please compare your results to past studies on similar cultures.

**Minor comments**

**First page**

- Dominion = Dominance?
- Conclusions: are you sure that these ants are detritivore?

**Introduction**

- Second sentence: please reduce the number of citations.
- Paragraph #2 (Similarly…) : please reduce the number of citations.

*Last paragraph:*

- Change to "this work focused..."
- Please describe the management of the plots (conventional/organic, tillage, pesticides, fertilization, irrigation and so on).
- Please replace the second sentence and the rest by a description of the method, period of sampling, taxa covered, taxonomical resolution. No need to give the structure of the paper.

**Methods**

- Please say in the text that sampling is based on litter and monolith extraction and is inspired by the TSBF method.
- *Table 1*: Please mention what these columns refer to.
- *Table 2*: Latitude and longitude are incorrectly formatted, with two commas in some cases, no commas in other cases. Please reformat with dots (".") as decimal separators and do not use other separators.
- Please describe the management of the plots (see comment above regarding the first page).
- The size of the sampling area is not clear. Please clarify.
- Was it really 4mm the smallest body size covered? Usually it is 1.5 mm for soil macrofauna.
- Please change "measured variables' by 'diversity components' or something similar.
- Please delete " (various population densities 1 m2)".
- Isn't it 'Dominance' instead of 'Domain'?
- No need to give the formula of the Shannon index.

**Results & Discussion**

- *Table 3 & 4:*
  - The title should be 'total and relative…by function groups in the different plots" Please use the unit Ind. m-2 for the absolute abundances.
  - Please add the standard deviation or the standard error.
- *Figure 2:* The name of the taxa is difficult to read because of the low quality of the plot.
- *Table 5:* These are taxa, not species. Please translate into ind.m-2, add errors, and remove totals by location.
- *Table 6:* "lumbrices" is not a correct genus or taxa, please correct.
- There are many typos in the name of family and genus. Please correct (e.g., Formocidae).
- Please compare your results with previous studies on banana plantations.
- *Table 7:* please explain what is "taxa_" and "individuals".
- *Table 8: *Please use "ind.m-2" not "x m2".
- Contrary to what you say, there are herbivores in the farm 8. Please correct.
- You can't link the absence of herbivore to the presence of spiders, based on correlation only. You need an experiment for that. Please remove this sentence.

**Diversity and productivity**

- Very interesting, but it is not clear if you did extra sampling for this topic. Please clarify. If so, please describe the sampling, if not, please explore the relationship with other taxa or functional groups.
- In any case, please explain how productivity estimates were related to soil fauna sampling: was it measured in each subplot (in this case you have one measure of productivity for one soil fauna monolith), or is it at a larger scale?
- *Table 9:*
  - Please clarify what you mean by earthworm production and add units.
  - The title of the last column is not clear, please clarify and provide the units.
- *Figure 3:* 
  - Please use worm and Hymenoptera for the x axis and the productivity for the Y axis.
  - What do you mean by "real"?
- In all the results sections, you should explain and use the difference among plots to explain the variations in soil macrofauna. For instance, don't you have soil data or management data?
- *Table 10:* please say "banana production".
- *Figure 3 & table 11:* 
  - If you have several replicates per farm, please use them in fig. 3 and add the regression line for all data and by plot, and include plot as a random factor in the analysis.
  - Consider changing table 11 by a pairwise correlation plot the tests on one side of the diagonal (see ggpairs() in R for instance).
- *Figure 4:* please add taxa richness and if possible functional groups' abundance.

**Data availability:**

- Please put the data from the two sites into one file.
- Please use the standard reporting template for soil macrofauna communities: https://zenodo.org/record/7691884
- Important: Please provide the raw data; by monolith or by layer, not aggregated by plot.
- Please also add metadata: coordinates, date of sampling (year, month, day), management, and so on (see the template).

Is the work clearly and accurately presented and does it cite the current literature?

Yes

If applicable, is the statistical analysis and its interpretation appropriate?

Partly

Are all the source data underlying the results available to ensure full reproducibility?

Partly

Is the study design appropriate and is the work technically sound?

Yes

Are the conclusions drawn adequately supported by the results?

Partly

Are sufficient details of methods and analysis provided to allow replication by others?

Partly

Reviewer Expertise:

Soil ecology

I confirm that I have read this submission and believe that I have an appropriate level of expertise to confirm that it is of an acceptable scientific standard, however I have significant reservations, as outlined above.

# Response

**Published:** 2023-08-11

**Authors:** C. A. Zuniga-Gonzalez

**Affiliations:** Agroecology. ECAV. CIByCC., National Autonomous University of Nicaragua, Leon. Bioeconomy and Climate Change Research Center., Leon, Leon, Nicaragua

Main comments

 [1] Please improve the English language and grammar.

 Response: Dear reviewer we appreciate your valuable observation, we are proceeding with the total revision of the article.

 [2] Please give more information about the differences between the plots and use them to explain the differences in soil macrofauna communities.

 Response: Thanks for this observation. In Methods section was added this information with the subtitle farms data where explicated your characteristics and differences.

 [3] Please compare your results to past studies on similar cultures. 

 Response: Yes, the past studies were compared with Costa Rica and Nicaragua studies.

 However, were added more cites as you indicated. Table 2 (Paragraph one) is similar to Zerbino *et al.* (2008), we also, refers the works of Priego-Castillo et al. (2009) and Castillo and Vera (2000) for the table 4, 5 and 6, also we refer to Pashanasi (2001), Zerbino (2010), the results of table 5 are referred and similar to Melo (2010), Rousseau et al. (2012, 2013). For the results of the Table 8 and 9 were added the cites.

 Response: Dear thanks for this observation, it was added. In each result was added cite as you indicated.

 Minor comments

 [4] First page Dominion = Dominance?

 Response: Thanks for it. We checked all the article and it was changed.

 [5] Conclusions: are you sure that these ants are detritivores?

 Response: Dear reviewer you know that Detritivorous ants are those that primarily feed on dead organic matter and help break down and recycle organic materials in ecosystems. We find two genera (earthworms and Hymenoptera), so the ants are referred to the detritivore genus because ants feeds on dead and decaying organic matter, such as plant material ( *Musa acuminata x Musa balbisiana (AAB)*, they are moist soils with a lot of plant organic matter. Thanks for the observation.

 Introduction

 [6] Second sentence: please reduce the number of citations. Paragraph #2 (Similarly…) : please reduce the number of citations.

 Response: Thanks for this observation, we have been checked all article and changed it.

 [7] Last paragraph: Change to "this work focused..."

 Response: It was changed.

 [8] Please describe the management of the plots (conventional/organic, tillage, pesticides, fertilization, irrigation and so on).

 Response: Thanks for this observation we have added a subsection (Agro system farms) in the methods section, there we explain the characteristics of the 8 farms and how they have been moving from conventional practices to agroecological farms.

 [9] Please replace the second sentence and the rest by a description of the method, period of sampling, taxa covered, taxonomical resolution. No need to give the structure of the paper.

 Response: Dear thanks for your observations, it was added in the last paragraph of the Introduction, second sentences as you indicated. We have added a cite of the protocol.oi where we explicate the methodology. Thanks for the observation.

 Methods

 [10] Please say in the text that sampling is based on litter and monolith extraction and is inspired by the TSBF method.

 Response: It was added. Also we added the cite [47].

 [11] Table 1: Please mention what these columns refer to.

 Response: Thanks for this observation, a comment on this was added before Table 1.

 [12] Table 2: Latitude and longitude are incorrectly formatted, with two commas in some cases, and no commas in other cases. Please reformat with dots (".") as decimal separators and do not use other separators.

 Response: Thanks for this observation. We made the changes. (e.g., 12°31'15.4"N Latitude 86°54'40.2"W Longitude)

 [13] Please describe the management of the plots (see the comment above regarding the first page).

 Response: Yes it was added as indicated in [8] item. Was elaborated a chart with your differences.

 [14] The size of the sampling area is not clear. Please clarify.

 Response: Ok thanks for this observation. The description of Tables 2 and 5 is explicating that the area is of 0.7 ha, and a total of 80 samples of macrofauna populations were identified, coded, and stored in the second phase. The study area in each farm or plot was 0.7 ha delimited 1 in 1,000 m ^2^ (50 cm long × 20 cm wide), as described in Rousseau et al. (2012), Rousseau et al. (2013), and Medina et al. (2021) . However, we added this area for each plot: The area where the sample was collected are: Farm1 246.5 ha, Farm2 14.1, ha, Farm3 67 ha, Farm4 7 ha, Farm5 4.2 ha, Farm6 2.1 ha, Farm7 3.5 ha, Farm8 4.9 ha. In total 80 samples were collected in 1000 m2 for 40 biomass samples and 40 edaphic samples in 0-20 cm depth.

 [15] Was it really 4mm the smallest body size covered? Usually, it is 1.5 mm for soil macrofauna.

 Response: Dear thanks for this observation, it was corrected.

 [16] Please change "measured variables' by 'diversity components' or something similar.

 Response: It was changed.

 [17] Please delete " (various population densities 1 m2)".

 Response: It was eliminated.

 [18] Isn't it 'Dominance' instead of 'Domain'?

 Response: It was changed.

 [19] No need to give the formula of the Shannon index.

 Response: Dear reviewer, thanks for this observation. Dear reviewer, we have decided to give the formula. 

 Results & Discussion

 [20] Table 3 & 4:

 - The title should be 'total and relative…by function groups in the different plots" Please use the unit Ind. m-2 for the absolute abundances.

 - Please add the standard deviation or the standard error.

 Response: Ok it was made in both tables.

 [21] Figure 2: The name of the taxa is difficult to read because of the low quality of the plot.

 Response: Ok we sent another figure with the best resolution.

 [22] Table 5: These are taxa, not species. Please translate into ind.m-2, add errors, and remove totals by location.

 Response: Ok thanks for this observation. All was changed.

 [23]Table 6: "lumbrices" is not a correct genus or taxa, please correct it.

  Response: Yes, it is correct, thanks for this observation. All was changed.

 [24] There are many typos in the name of family and genus. Please correct (e.g., Formocidae).

  Response: Ok thanks for this observation. It was checked and corrected.

 [25] Please compare your results with previous studies on banana plantations.

  Response: Of course, it was checked and added as you indicated.

 [26] Table 7: please explain what is "taxa_" and "individuals".

  Response: Thanks for this observation, the taxa was change by Genus Types and individuals, by Ind. m ^2^

 [27] Table 8: Please use "ind.m-2" not "x m2".

 Contrary to what you say, there are herbivores in the farm 8. Please correct.

 You can't link the absence of herbivore to the presence of spiders, based on correlation only. You need an experiment for that. Please remove this sentence.

 Response: Ok thanks for this observations. It was corrected.

 [28] Diversity and productivity

 Very interesting, but it is not clear if you did extra sampling for this topic. Please clarify. If so, please describe the sampling, if not, please explore the relationship with other taxa or functional groups.

 In any case, please explain how productivity estimates were related to soil fauna sampling: was it measured in each subplot (in this case you have one measure of productivity for one soil fauna monolith), or is it at a larger scale?

 Response: Dear reviewers thanks for this observation. The sampling is the same that was recollected, but in this moments of the analysis we compare the populations of the Hymenoptera (Insects) and Oligochaeta (Worm or Earthworm) that were the more representative in this study. So, we the Pearson correlation coefficient that is a measure of linear correlation between two sets of data in this case the Agricultural production (banana units per ha) and the abundance of *Earthworms* and hymenopteran insects (Ind. m ^2^). It is the ratio between the covariance of two variables and the product of their standard deviations.

 [29] Table 9:

 - Please clarify what you mean by earthworm production and add units.

 Response: Thanks, we refer the abundance of Earthworms and the unit Ind. m ^2^ was added.

 - The title of the last column is not clear, please clarify and provide the units.

 Response: Thanks, It was change by Abundance Estimated overall Earthworm production

 Ind. m ^2^.

 [30] Figure 3:

 - Please use worm and Hymenoptera for the x axis and the productivity for the Y axis.

 - What do you mean by "real"?

 Response: Dear is ok the change was made. The Real is the production of Banana or plantains, during the periods studied. Measured in Units by ha. Dear reviewer by best interpretation all was change by **Production (Units ha**).

 [31] In all the results sections, you should explain and use the difference among plots to explain the variations in soil macrofauna. For instance, don't you have soil data or management data?

 Response: Dear all results section was checked and added the comments that you indicated. Regarding to the management data the farmer don’t had information because the cost to get it (Laboratory of soil).

 [32] Table 10: please say "banana production".

  Response: OK

 [33] Figure 3 & table 11:

 - If you have several replicates per farm, please use them in fig. 3 and add the regression line for all data and by plot, and include plot as a random factor in the analysis.

 - Consider changing table 11 by a pairwise correlation plot the tests on one side of the diagonal (see ggpairs() in R for instance).

  Response: Dear reviewer thanks for your observation but our aim was to relate the plots versus the genus identified, however, yes we go to make o add the table that you suggest us as Fig 5. Dear, we appreciate your observation, but due to a problem that we had already defined in the methodology that we work with SPSS, which suggests that we do it with SPSS and we have added fig. 6.

 [34] Figure 4: please add taxa richness and if possible functional groups' abundance.

 Response: Dear thanks for this observation, only we added taxa richness, the functional group’ abundances, we consider that was discussed on the table 3 and 4. Was added the Fig 5. Shannon Index.

 [35] Data availability:

 Please put the data from the two sites into one file.

 Response: Ok

 Please use the standard reporting template for soil macrofauna communities: https://zenodo.org/record/7691884

 Important: Please provide the raw data; by monolith or by layer, not aggregated by plot.

 Please also add metadata: coordinates, date of sampling (year, month, day), management, and so on (see the template).

 Response: Ok thanks for your consideration.

# Response

**Published:** 2024-01-10

**Authors:** C. A. Zuniga-Gonzalez

**Affiliations:** Agroecology. ECAV. CIByCC., National Autonomous University of Nicaragua, Leon. Bioeconomy and Climate Change Research Center., Leon, Leon, Nicaragua

Dear Reviewer 2, the data availability, has been duly addressed:

1. **Data Compilation**: The data from both sites has been consolidated into a singular file as requested.
2. **Template Utilization**: The standard reporting template for soil macrofauna communities, accessible at https://zenodo.org/record/7691884, has been utilized to format the dataset.
3. **Data Format**: The provided data remains in its raw form, organized either by monolith or by layer, ensuring it is not aggregated by plot, as specified.
4. **Metadata Inclusion**: Essential metadata such as coordinates, sampling date (year, month, day), management details, and other relevant information, following the template, have been added to the dataset.

By adhering to these instructions, we've ensured the data is compiled appropriately, formatted using the standard template, remains in its raw form, and includes essential metadata, thereby meeting the specified requirements for data availability.

# Reviewer report: Reviewer response for version 1

**DOI:** 10.5256/f1000research.139795.r155921

**Published:** 2023-03-07

**Authors:** Angel Sol-Sánchez

**Affiliations:** 1 Campus Tabasco, Colegio de Postgraduados, Texcoco, State of Mexico, Mexico

The article is interesting and important in the banana production activity.

1. *"The first was.."* - must say 'the first one'.
2. In the results, the correct way is 'ind/m ^2^'. Example: 160 ind/m ^2^.
3. *"In four farms (San Martín, Santa Isabel and El Verdon)…"* and which one is the last?
4. Incorrect to cite too many authors: *"These data are supported by Zayas et al. (2022), Quiroz-Medina et al. (2021), Castillo y Vera (2000), Pashanasi (2001), Zerbino (2010), Rousseau et al. (2012) and Rousseau et al. (2013)." - *Maybe you could cite just three authors together in the text; because information is the same.
5. It is not a conclusion, but a result: *"The diversity and richness of the edaphic macrofauna was evaluated in eight farms in the western area. A total of 78.72% of the individuals were identified in the soil from 0–20 cm ^2^, while the remaining 21.26% in the foliage. In the first four farms in the León area, 23 genera were found, and in the Posoltega farms, 21 genera. The relative abundance of León was 1,450 individuals per m ^2^, while in the Posoltega area it was 1,700 individuals per m ^2^."*
6. Bibliography incomplete as: Ruiz N, Lavelle P, Jiménez J: SOIL MACROFAUNA FIELD MANUAL.2008; p. 113.

Is the work clearly and accurately presented and does it cite the current literature?

Yes

If applicable, is the statistical analysis and its interpretation appropriate?

Yes

Are all the source data underlying the results available to ensure full reproducibility?

Yes

Is the study design appropriate and is the work technically sound?

Yes

Are the conclusions drawn adequately supported by the results?

Partly

Are sufficient details of methods and analysis provided to allow replication by others?

Yes

Reviewer Expertise:

Climate change, Ecology, Mangroves and tropical forestry

I confirm that I have read this submission and believe that I have an appropriate level of expertise to confirm that it is of an acceptable scientific standard.

# Response

**Published:** 2023-03-07

**Authors:** C. A. Zuniga-Gonzalez

**Affiliations:** Agroecology. ECAV. CIByCC., National Autonomous University of Nicaragua, Leon. Bioeconomy and Climate Change Research Center., Leon, Leon, Nicaragua

I appreciate the comments of the reviewer1 and I confirm and accept the improvements to be incorporated.

# Response

**Published:** 2023-08-08

**Authors:** C. A. Zuniga-Gonzalez

**Affiliations:** Agroecology. ECAV. CIByCC., National Autonomous University of Nicaragua, Leon. Bioeconomy and Climate Change Research Center., Leon, Leon, Nicaragua

The article is interesting and important in banana production activity.

 Dear Reviewer, thanks for this.  

1. *"The first was.."* - must say 'the first one'. 
  Response: It was improvements. 
2. In the results, the correct way is 'ind/m ^2^'. Example: 160 ind/m ^2^. 
  Response:  Dear reviewer all the results were checked and improvements. 
3. *"In four farms (San Martín, Santa Isabel and El Verdon)…"* and which one is the last?
  Response: The Quinta Cony was added, thanks for this observation. 
4. Incorrect to cite too many authors: *"These data are supported by Zayas et al. (2022), Quiroz-Medina et al. (2021), Castillo y Vera (2000), Pashanasi (2001), Zerbino (2010), Rousseau et al. (2012) and Rousseau et al. (2013)." - *Maybe you could cite just three authors together in the text; because information is the same. 
  Response:  It was correcting that only 3 authors were added, but in this case was putting 4. 
5. It is not a conclusion, but a result: *"The diversity and richness of the edaphic macrofauna was evaluated in eight farms in the western area. A total of 78.72% of the individuals were identified in the soil from 0–20 cm ^2^, while the remaining 21.26% in the foliage. In the first four farms in the León area, 23 genera were found, and in the Posoltega farms, 21 genera. The relative abundance of León was 1,450 individuals per m ^2^, while in the Posoltega area it was 1,700 individuals per m ^2^.".  *
  Response: Dear reviewer it was translated to the results section, thanks for this observation. 
6. Bibliography incomplete as: Ruiz N, Lavelle P, Jiménez J: SOIL MACROFAUNA FIELD MANUAL.2008; p. 113. 
  Response: It was corrected and added the link  https://hal-bioemco.ccsd.cnrs.fr/bioemco-00560386 and the Bioemco as a source. 

Dear reviewer, thanks for all your observations.
